# “You're Only as Strong as Your Weakest Link”: A Current Opinion about the Concepts and Characteristics of Functional Training

**DOI:** 10.3389/fphys.2017.00643

**Published:** 2017-08-30

**Authors:** Cauê V. La Scala Teixeira, Alexandre L. Evangelista, Jefferson S. Novaes, Marzo E. Da Silva Grigoletto, David G. Behm

**Affiliations:** ^1^Faculty of Physical Education, Praia Grande College São Paulo, Brazil; ^2^Studies Group of Obesity, Interdisciplinary Laboratory of Metabolic Diseases, Federal University of São Paulo São Paulo, Brazil; ^3^Department of Education, Nove de Julho University São Paulo, Brazil; ^4^Department of Gymnastics, Physical Education Graduate Program, Federal University of Rio de Janeiro Rio de Janeiro, Brazil; ^5^Department of Physical Education, Federal University of Sergipe Aracaju, Brazil; ^6^School of Human Kinetics and Recreation, Memorial University of Newfoundland St. John's, NL, Canada

**Keywords:** integrated training, multicomponent training, hybrid training, multimodal training, task-specific training

## Introduction

In the twenty-first century, functional training (FT) has become a strong worldwide fitness trend (Thompson, 2016), resulting in a growing interest to investigate its effects on many variables (e.g., morphological, physiological, and psychological) with different populations (children, adults, and elderly). Confirming this view, the current position stand of the American College of Sports Medicine on the prescription of physical exercise for healthy individuals includes FT (also termed: “neuromotor training”) as one of the modalities to be considered (Garber et al., 2011).

Although the tools (exercises, equipment, and accessories) used in current functional training have long been employed in rehabilitation and conditioning programs, the systematic use of these tools, as well as scientific interest in this topic, are recent phenomena (Anderson and Behm, 2004; Rhea et al., 2008; Gordon and Bloxham, 2016). However, since it is still a subject of recent scientific interest, there are many methodological conflicts and divergences in training prescriptions (La Scala Teixeira et al., 2016). For example, some studies have associated FT with the use of instability and applied unstable bases in many exercises (Pacheco et al., 2013), while other studies have used instability in a small part of exercises (Weiss et al., 2010; Distefano et al., 2013) or have not used any unstable bases (Lohne-Seiler et al., 2013).

In view of these considerations, detailing the actual concepts and characteristics of FT forms the basis for maximizing the benefits of both research and day-to-day interventions in terms of performance or rehabilitation (Behm et al., 2010). However, the methodological divergence observed with practical interventions in several fitness facilities, as well as in scientific studies, points to a reality in which the real concept of FT and all that it encompasses are still not well-elucidated (Fowles, 2010).

A major factor that has contributed to this problem in the general population are probably the marketing campaigns promoting FT, which explore random several medias in order to attract consumers (Da Silva-Grigoletto et al., 2014). For example, publications of functional exercises can contain at the same time exercises of low (e.g., planks and squats) and high complexity (e.g., Olympic weightlifting and calisthenics/gymnastics exercises). Similarly, marketing explores simple and low-cost accessories (e.g., balls, balance disk, elastic bands, medicine balls), as well as expensive equipment (e.g., multi-station machines, pneumatic resistance equipment). Although contributing to the consolidation of the term “functional training” in the fitness scenario, this wide variation in publications impairs consolidation of its true concepts and characteristics (La Scala Teixeira and Evangelista, 2014).

Taken together, these facts highlight the need for researchers to establish a consensus about the concept of FT so that studies can be conducted according to a methodological pattern using pre-established criteria and, finally, that coaches and practitioners can make practical applications based on sound theoretical and scientific evidence. Therefore, in this paper we defined the concepts and characteristics of FT based on the analysis of current and relevant specific technical and scientific literature.

## Functional training: conceptual basis

The word “training” is related to a set of exercises performed by a person with the intention to improve a specific skill (Reilly et al., 2009). “Functional” is associated with daily function, which is performed in an attempt to satisfy a function and to meet a practical goal. Hence, the combination of the two words refers to the understanding that FT is a set of exercises performed by a person with the intention of improving performance in daily functions. Daily functions are defined as all activities of daily living (ADLs) frequently performed by humans, which range from the simple maintenance of posture in static positions to more complex activities such as walking, pushing, pulling, squatting, and rolling (Okada et al., 2011).

To understand the concept of FT, one should first analyze the characteristics of ADLs. Among the different characteristics observed, one that is common to all (or almost all) ADLs is the need to simultaneously use different physical capacities (Moran et al., 2016). For example, the act of walking, as simple as it may seem, depends on the combined and simultaneous use of different physical capacities such as strength, mobility/flexibility, balance, motor coordination, and postural stability (Okada et al., 2011). If the same activity is maintained over a prolonged period, muscle endurance and cardiorespiratory fitness are added to the other capacities. Thus, the basic premise of FT is the application of exercises that stimulate the development, preferably simultaneous, of these physical capacities (Okada et al., 2011).

In agreement with this idea, two recent studies concluded that the concept of FT combines this type of training with the aim of developing different physical capacities in an integrated and balanced manner to ensure efficiency (good performance with low energy expenditure) and safety (low risk of injury) during ADLs, work or sports tasks, respecting the biological and methodological principles of training, especially biological individuality and specificity (La Scala Teixeira and Evangelista, 2014; La Scala Teixeira et al., 2016). According to training specificity principle, the training activities/program should mimic as closely as possible the athletic or work activity (Behm and Sale, 1993). FT specificity should reflect movement velocity, contraction types (i.e., concentric, eccentric, or isometric), and intensities (strength vs. endurance needs), joint angles, balance challenges, range of motion, and other applicable capacities. Supporting the above, Thompson (2016) defines FT as the use of strength training not only to develop strength, but also balance, motor coordination, power and endurance, increasing the ability of individuals to execute ADLs, whether they be simpler tasks of daily living or more complex athletic maneuvers.

This concept of integration has been explored in most scientific studies related to FT; however, different authors and research groups have adopted different terms for a clearer representation of the concept. It has even been suggested that the term FT is no longer the most appropriate (Da Silva-Grigoletto et al., 2014). In this respect, other terms are used as conceptual synonyms of FT such as integrated training (Distefano et al., 2013), multicomponent training (Bouaziz et al., 2016), hybrid training (Liu et al., 2014), multimodal training (Thompson and Osness, 2004), and task-specific training (Manini et al., 2007). Although the terms are different, the idea of integration is implied in all of the terms.

**Figure 1:**
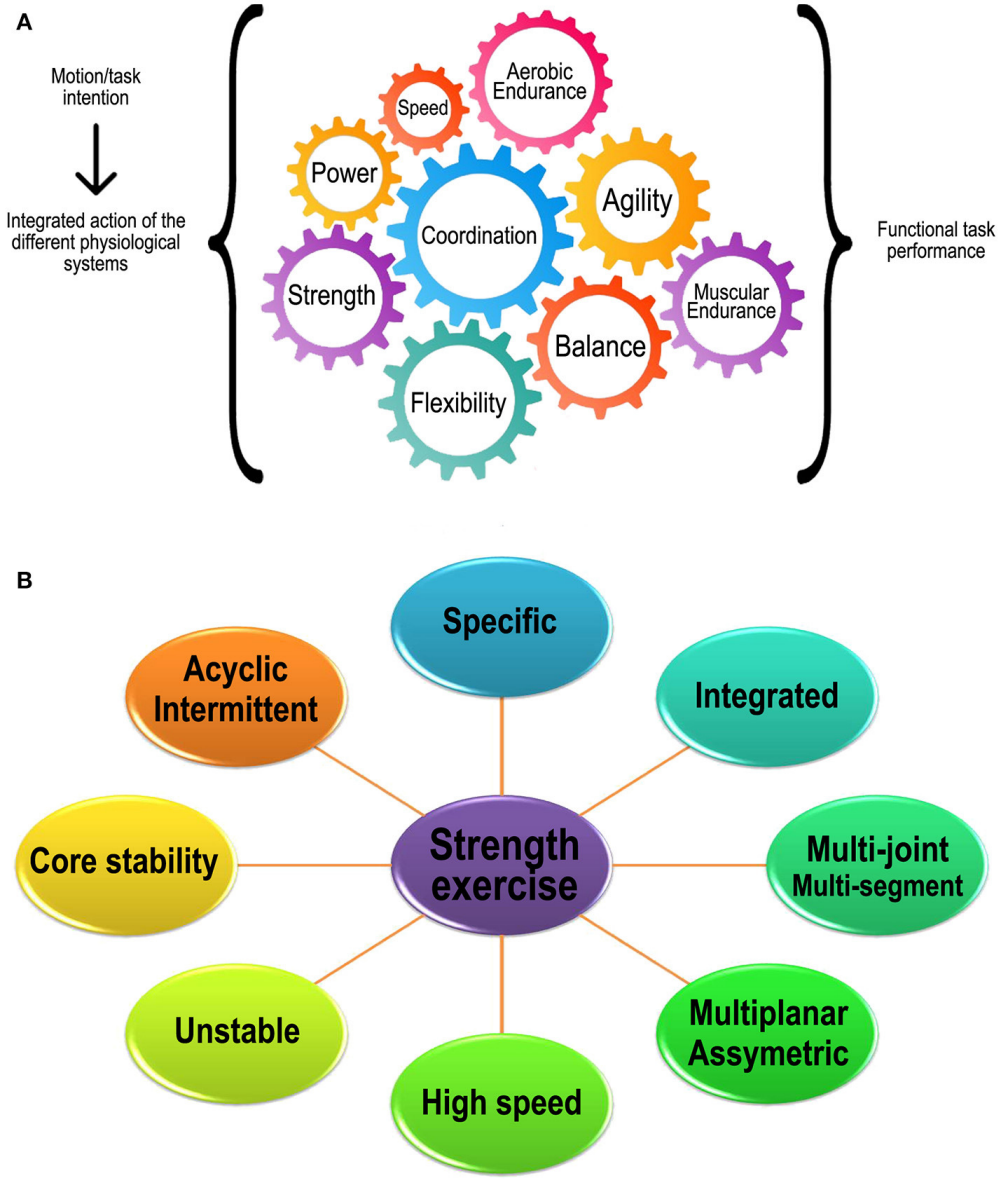
**(A)** Schematic representation proposal of the integration of physical capacities/physiological systems during the execution of functional tasks; theoretical basis for the conceptual construction of FT; **(B)** Schematic representation of the characteristics of functional training/exercise.

Finally, D'Elia (2013) summarizes the importance of the concept of FT in the phrase “you are only as strong as your weakest link.” This phrase refers to the idea that functionality results from the efficiency of an integrated system, like a gear train in which each gear corresponds to one physical capacity. In this system, if one gear fails, the function of the whole system is compromised, thus demonstrating the importance of the integrated and balanced development of different physical capacities (Figure 1A).

## Characteristics of functional training

The main characteristic of FT is the integration of physical capacities/physiological systems, functioning as a kind of combined and/or integrated training (Distefano et al., 2013). However, in traditional combined training, two or more physical capacities are trained in the same session (e.g., strength and endurance) but at different times or with different exercises, while in FT these capacities are trained simultaneously, preferentially in the same exercise. This characteristic is related to the specificity of training (Behm and Sale, 1993), i.e., the need to provide stimuli resembling ADLs. Thus, specificity is another characteristic of FT (Liu et al., 2014; La Scala Teixeira et al., 2016).

Considering the need to be specific, the previous analysis of ADLs permits the identification of some additional characteristics that are included in FT programs/exercises. It should be remembered that all of these characteristics are directly related to the concept of integrated and balanced development of different physical capacities (Figure 1B). One of these characteristics is the application of multi-joint movements not only to exercises that involve the upper or lower limbs (e.g., bench press and leg press), but that require actions involving all body segments (multi-segment) in an integrated and simultaneous manner (e.g., clean and thruster) (Heinrich et al., 2012). In addition to resembling ADLs, these movements raise the level of stress to the neuromuscular system, stimulating the development of strength, power, motor coordination, balance, and flexibility/mobility (Hedrick and Wada, 2008).

Another characteristic observed in ADLs and used in FT is the possibility to execute asymmetric movements applying lateral weight shift (e.g., lateral squat), unilateral, or alternated tasks (e.g., lunges) and movement in multiple planes (functional diagonal movements; Weiss et al., 2010; Gonzalo-Skok et al., 2016). In addition to improving strength, these movement stimulate motor coordination, mobility, balance (Bishop et al., 2016) and agility (Gonzalo-Skok et al., 2016).

One more important aspect to be highlighted is the fact that, in ADLs, the speed of movement is variable and is generally fast. For example, the movement of rising from a chair tends to occur within fractions of a second (Rikli and Jones, 1999). Thus, FT incorporates higher velocities of execution (e.g., <1 second per phase) (Distefano et al., 2013; Cadore et al., 2014), increasing the requirement of acceleration and deceleration and stimulating both type I and II muscle fibers. This is an important stimulus for physical capacities such as strength and power, as well as motor coordination, agility, speed, proprioception, and balance (Lohne-Seiler et al., 2013).

Another characteristic observed in ADLs and also incorporated in FT is the execution of non-cyclic tasks, i.e., the sequential execution of different movements/actions (e.g., rising from a chair, walking a short distance, climbing a flight of stairs, sitting again). To apply this characteristic, FT programs generally adopt circuit training (Pacheco et al., 2013; Neves et al., 2015) since this approach permits the sequential execution of different tasks, providing greater challenge to the neuromuscular system and stimulating cognitive capacity. Furthermore, the execution of non-cyclic tasks is also characterized by intermittent effort (periods of high- intercalated with periods of low-intensity or absolute rest). In this respect, FT also explores intermittent stimuli in which some protocols of high-intensity interval training are used with a certain frequency (McRae et al., 2012; Neves et al., 2014), stimulating the development of cardiorespiratory fitness.

Instability is another feature of ADLs, which is characterized by perturbation of the position of the center of gravity during static and dynamic tasks. Although exposure to unstable bases is not common in everyday life, instability is common, due to the action of extrinsic and intrinsic forces on the body associated with frequent alterations in the area and position of the support base (McBride et al., 2006). For example, perturbation of the position of the center of gravity occurs during walking due to the alternation of steps, weight transfer from one leg to the other leg and variation in the base of support, increasing the levels of instability. Thus, FT also incorporates instability through the use of unstable bases (Rabay et al., 2012), stable bases but with a small surface of support and/or variable surfaces (Pacheco et al., 2013), unstable loads (Kohler et al., 2010), and exercises performed in a standing position (Balachandran et al., 2016). These tools stimulate strength development concomitantly with the development of balance, motor coordination, and body/postural perception (Behm and Colado, 2012; Behm et al., 2015). However, instability devices are not always essential for promoting optimal strength and balance, specially to athletes (Behm et al., 2010).

Instability resistance training characteristics (i.e., decreased force and power output) are not optimal for strength or power training but should be favorable for rehabilitation (Behm and Colado, 2012, 2013). The instability-induced decrease in force and power output can still provide a healthy stress on a recovering joint or muscle (Behm and Colado, 2012). Vertebral joint stiffness occurs with contractions as low as 25% MVC (Cresswell and Thorstensson, 1994). Since the efficiency of the multifidus can be improved with training loads of 30–40% of MVC (Cholewicki and McGill, 1996), the lower force outputs are still suitable for back rehabilitation, while the increased trunk and limb muscle activation provide greater stabilization. The Behm and Colado's (2012) review reported 47.3% increase in trunk stabilizer muscle activation (effect size: 2.48). Training the core musculature improves the robustness of the stabilizing system, protecting against low back injuries (Reeves et al., 2007). Furthermore, instability-induced coordination challenges could improve motor control adaptations (i.e., co-activations, anticipatory postural adjustments) (Behm and Colado, 2012). The Canadian Society of Exercise Physiology Position stand states, “Individuals who are involved with rehabilitation, health-related fitness pursuits or cannot access or are less interested in the training stresses associated with ground based free weight lifts, can also receive beneficial resistance training adaptations with instability devices and exercises to achieve functional health benefits” (Behm et al., 2010, p. 111).

In the ADLs, dual tasks are also frequently observed. Dual tasks consists of performing two tasks at the same time, usually a motor action and a cognitive task simultaneously. Think of the daily appointments or mentally plan the route while walking is a common example of dual task. Thus, FT also incorporates the dual task as one of the characteristics that can be explored in the exercises, especially in the elderly (Granacher et al., 2011; Cadore et al., 2014). In the exercise training, the dual task can increase functional performance in everyday dual task situations (e.g., walking and counting the months of the year), as well as reducing the risk of falls in the elderly (Granacher et al., 2011).

Finally, to ensure the efficiency and safety during the execution of static and dynamic everyday tasks, it is necessary to maintain a good posture pattern, especially in the core region (McGill, 2010). Consequently, the focus on developing core stability is a marked feature of FT programs (Tomljanović et al., 2011; Heinrich et al., 2012). For this purpose, the practical application of the aforementioned characteristics (multi-joint/multi-segment, asymmetric, and multi-planes exercises executed at high speed, instability) is already sufficient to provide stimuli for the improvement of core stability, which directly influences proprioception, body/postural perception, balance, agility and strength production.

## Conclusion

The concept of FT is related to development of different physical capacities in an integrated and balanced manner in order to provide autonomy, efficiency and safety during activities related to daily living, work and/or sports. For this purpose, FT uses strength exercises generally characterized by integrated, multi-joint/multi-segment, asymmetrical, multi-planes, acyclic, intermittent, speedy, and unstable movements that emphasize core stability.

## Author contributions

Substanial contributions to the conception and design of the work: CL, AE, JN, MD, and DB. Draft the work and revisit it critically for important intellectual content: CL, AE, JN, MD, and DB. Final approval of the version to be submitted and published: CL, AE, JN, MD, and DB. Agreement to be accountable for all aspects of the work in ensuring that questions related to the accuracy and integrity of any part of work are appropriately investigated and resolved: CL, AE, JN, MD, and DB.

### Conflict of interest statement

The authors declare that the research was conducted in the absence of any commercial or financial relationships that could be construed as a potential conflict of interest.
